# A Novel Method for Inserting Dose Levels Mid‐Trial in Early‐Phase Oncology Combination Studies

**DOI:** 10.1002/sim.70417

**Published:** 2026-02-12

**Authors:** Matthew George, Ian Wadsworth, Pavel Mozgunov

**Affiliations:** ^1^ Phastar London UK; ^2^ MRC Biostatistics Unit University of Cambridge Cambridge UK

**Keywords:** adaptive designs, combination trials, dose‐finding, dose insertions

## Abstract

The use of combination treatments in early‐phase oncology trials is growing. The objective of these trials is to search for the maximum tolerated dose combination from a predefined set. However, cases in which the initial set of combinations does not contain one close to the target toxicity pose a significant challenge. Currently, solutions are typically ad hoc and may bring practical challenges. We propose a novel method for inserting dose levels mid‐trial, which features a search for the contour partitioning the dose space into combinations with toxicity truly above and below the target toxicity. Establishing this contour with a degree of certainty suggests that no combination is close to the target toxicity, triggering an insertion. We examine our approach in a comprehensive simulation study applied to the PIPE design and two‐dimensional Bayesian logistic regression model (BLRM), though any model‐based or model‐assisted design is an appropriate candidate. Our results demonstrate that, on average, the insertion method can increase the probability of selecting combinations close to the target toxicity, without increasing the probability of subtherapeutic or toxic recommendations.

## 1 Introduction

Dose‐finding is a key step in the clinical development of a new treatment, and the choice of early‐phase design can have a profound impact on success in later phase trials [1]. Phase I trials typically aim to find the highest dose that can be administered while ensuring patients are at a low risk of serious side effects. To offer a higher chance of successful treatment in oncology, under the assumption that efficacy increases monotonically with dose level, there is a willingness to accept a dose that leads to some toxic responses, commonly called dose‐limiting toxicities.

Various approaches have been used to guide the dose‐finding process in the single‐agent setting. These can be categorized into the following three types of design. Rule‐based designs, such as the 3 + 3 design, provide the simplest approach available. However, a number of publications have cited significant disadvantages with the 3 + 3 design [1, 2, 3]. The emergence of Project Optimus, an initiative developed by the Oncology Center of Excellence, further highlights the need for more effective strategies in the dose‐finding and dose optimization paradigm across oncology [4].

Model‐based designs use a parametric function to model the dose‐toxicity relationship, which in turn guides escalation decisions. Examples include the continual reassessment method (CRM) [5] and Bayesian logistic regression model (BLRM) [6]. Model‐free designs are often viewed as practical alternatives to model‐based designs, relying on some predefined rules to guide escalation, without directly modeling the dose‐toxicity relationship. Examples of these include the Bayesian Optimal Interval (BOIN) design [7] and the modified Toxicity Probability Interval (mTPI‐2) design [8]. Numerous simulation studies have demonstrated the effectiveness of various model‐based and model‐free designs over traditional rule‐based approaches [3, 9, 10].

The use of combination treatments in early‐phase oncology trials is growing [11, 12]. The objective of these trials is to search for the maximum tolerated dose combination, the combination with a probability of toxic response closest to the target level. One important difference in this setting is that the dose‐finding design must account for the order of toxicity being unknown for certain combinations. Many rule‐based, model‐based, and model‐free designs have been proposed, or extended from their single‐agent counterpart, to account for this additional challenge. The partial ordering continual reassessment method (poCRM) identifies all possible known toxicity orderings, simplifying the dose‐finding problem to a series of one‐dimensional CRM trials [13]. In addition, the design has been extended and applied in a three‐dimensional dose‐finding oncology trial [14]. The two‐dimensional BLRM uses a five‐parameter function to describe the relationship between each dose level and the joint toxicity probability [15]. The BOIN design has also been extended to handle two experimental treatments [16].

Novel methodology covering other, more complex settings is also available, and has been applied in practice in some cases. Dose‐finding trials in which one dose level is treated as continuous are supported by various designs [17, 18]. Mozgunov et al. propose a flexible design which handles a continuous efficacy endpoint [19]. Mozgunov et al. apply a Bayesian model‐based design for a trial with standard of care and an experimental immunotherapy treatment [20]. Applications of designs incorporating both toxicity and efficacy endpoints to guide escalation are also available in the literature [21, 22].

The focus of our paper is on combination designs. One consistent theme with existing combination methods is that they require a set of combinations to be specified before the dose‐finding process begins. Compounded by the limited amount of clinical data available in early phases, there is a considerable risk of designing a trial in which none of the prespecified combinations have a probability of toxic response close to the target level. Solutions to this problem in real trials are typically ad hoc, posing practical challenges. These may include delays (and cost implications) to allow for decision‐making and protocol updates, allocating too many subjects to subtherapeutic and/or toxic combinations before an insertion, and uncertainty around which combinations to insert (which can lead to a poorly characterized selection for later phases).

There are a few publications in the single‐agent setting which recognize this potential issue and suggest solutions. Firstly, Hu et al. [23] give a formal way to insert doses mid‐trial, driven by a quadratic logistic model, after identifying when none of the original doses are close to the target toxicity. However, their method is subject to bias should the underlying parametric model be misspecified [24]. Chu et al. [24] propose the adaptive dose modification method, in which they partition the dose‐toxicity space into three regions: underdosing, acceptable, and overdosing. Dose insertions are made when no doses belong to the acceptable region, and a polynomial regression model determines the new dose level. The flexibility, in the sense that the method could be applied to any design, is a significant positive. However, while their simulation study evaluates the proportion of trials selecting an inserted dose, there is no commentary on whether the inserted doses have acceptable toxicity.

In the binary outcome setting, there is some literature evaluating dose‐insertion methods. In the single‐agent setting, Guo et al. [25] present a method to insert doses considering both toxicity and efficacy outcomes. In the dual‐agent setting, Lyu et al. [26] propose their triple adaptive Bayesian design. This incorporates a dose‐insertion step, in which the biologically optimal dose combination is estimated via a utility function based on toxicity and efficacy, and the distance between this and existing combinations will dictate insertion decisions.

Importantly, practical solutions in the combination space have not been explored in the literature, particularly when toxicity is the primary outcome of interest. The objective of this paper is to propose a novel insertion method that addresses this gap. While many early‐phase dose‐finding trials use flexible protocol language that allows data‐driven dose insertions, our proposal offers an efficient and formal solution. The method involves searching for the contour, partitioning the dose combination space into combinations with toxicity truly above and below the target toxicity, based on data observed to that point. If we identify this contour with a degree of certainty, it suggests no existing combination is close to the target toxicity, triggering a dose insertion.

The paper continues as follows. Section 2 describes our novel insertion methodology. Section 3 provides a review of the underlying dose‐escalation designs we consider in this paper. Section 4 outlines our comprehensive simulation study and examines the results, which in particular show that our method yields more desirable trial outcomes when no starting combinations are close to the target toxicity. Section 5 guides us through an application of our method to a real trial, before Section 6 gives some concluding remarks.

## 2 Insertion Methodology

### 2.1 Setting

The insertion method we propose is appropriate for dose‐finding trials in which patients receive two active treatments in combination, where the objective is to establish the maximum tolerated dose combination. Specifically, this is the combination with probability of toxic response closest to the target level, θ.

We now formally define this framework. Suppose our trial has treatments Agent A and Agent B. There are I dose levels available of Agent A, denoted $d_{1}^{A},\ldots ,d_{I}^{A}$ and J dose levels of Agent B, denoted $d_{1}^{B},\ldots ,d_{J}^{B}$. Let $d_{ij}$ represent the combination of doses $d_{i}^{A}$ and $d_{j}^{B}$ for i=1,…,I and j=1,…,J. The total number of patients treated with combination $d_{ij}$ and who experience a toxic response receiving $d_{ij}$ are denoted $n_{ij}$ and $y_{ij}$ respectively. The toxicity probability on $d_{ij}$ is written as $\pi_{ij}$.

Under the Bayesian framework, each $\pi_{ij}$ can be modeled independently and assigned a beta prior $\pi_{ij}∼\text{Beta}(a_{ij},b_{ij})$ for hyperparameters $a_{ij}$ and $b_{ij}$ ∀i,j. Priors can be prespecified if knowledge of the toxicity of combinations is available. Alternatively, hyperparameters yielding noninformative priors can be chosen in the absence of prior knowledge on the dose‐toxicity relationship. Assuming each patient is independent such that $y_{ij}∼\text{Binomial}(n_{ij},\pi_{ij})$ ∀i,j, the posterior distribution for $\pi_{ij}$ can be written as

(1) $$\pi_{ij}|n_{ij},y_{ij}∼\text{Beta}(n_{ij}+a_{ij},n_{ij}-y_{ij}+b_{ij}).$$

An advantage of modeling each $\pi_{ij}$ independently is that it allows for easy identification of sharp increases in the toxicity between adjacent combinations. This is irrespective of the underlying dose‐escalation design.

Formally, the trial objective is to establish the combination $d_{ij}$ with corresponding probability of toxic response $\pi_{ij}$ closest to the target toxicity θ.

### 2.2 Proposed Method

The purpose of our proposed insertion method is to improve trial outcomes when none of the prespecified combinations have a probability of toxic response close to the target toxicity θ. Note that the method is independent of the underlying model driving escalation decisions, so it can be applied to any model‐based or model‐assisted design. The method harnesses the idea of searching for the maximum tolerated contour, $MTC_{\theta }$, introduced in the PIPE design [27]. This is the line partitioning the dose combination space into combinations with toxicity truly above and below θ. If a single eligible contour is identified as the $MTC_{\theta }$ with high probability given the current data, this suggests no existing combination has toxicity probability close to θ, motivating the insertion of new dose levels.

Suppose the underlying dose‐escalation design assigns a new cohort to $d_{ij}$, resulting in changes to $n_{ij}$ and $y_{ij}$. The following steps are carried out independently of the dose‐escalation design (since only the posteriors for each $\pi_{ij}$ drive insertions), and prior to the design considering any escalation decisions. This is advantageous for model‐based designs whose parametric form may not be flexible enough to model sharp increments in toxicity adequately. Firstly, we update our knowledge on $\pi_{ij}$ by recalculating its posterior from Equation (1). Secondly, the $MTC_{\theta }$ is re‐estimated, which will be one of the eligible contours. A description of how to define the eligible contours follows.

Each contour can be represented by a binary matrix, where entries are 0 or 1 depending on whether estimates of the probability of toxic response for a combination are below or above the contour, respectively. The monotonicity assumption limits the total number of possible contours to I+JI. Let ϑ be the set of all monotonic contours for an I×J dose combination space and define $C_{s}\in \vartheta$ as the binary matrix representing the contour s=1,…,I+JI.

In the 3×3 combination space, there are a total of 20 eligible contours, each of which is shown in Table 1. The top‐left matrix represents the case where all $d_{ij}$ are below the contour (all entries are assigned 0). The penultimate matrix represents the case where $d_{11}$ is below the contour (assigned 0) and all other $d_{ij}$ are above the contour (assigned 1s).

**TABLE 1:** Binary matrices representing all 20 eligible contours in the 3×3 combination space. Entries are 0 or 1 depending on whether estimates of the probability of toxic response for a combination are below or above the contour, respectively.

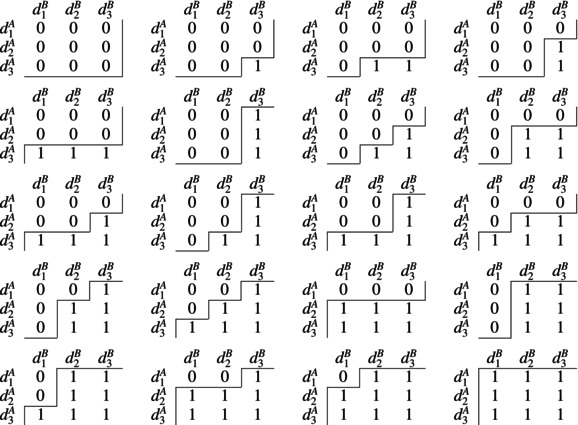

Now that we have defined our set of contours ϑ, we now describe how to search for which of these is our current estimate of the $MTC_{\theta }$. Given the current data, first calculate the posterior probability of each $\pi_{ij}$ being less than or equal to θ, that is

(2) $$p_{ij}(\theta |n_{ij},y_{ij})=\mathbb{P}(\pi_{ij}\leq \theta |n_{ij},y_{ij},a_{ij},b_{ij})$$

where the right‐hand side of Equation (2) is equal to the cumulative distribution function of a Beta distribution. Equation (3) gives the general formula for calculating the probability that the $MTC_{\theta }$ is defined by the matrix $C_{s}$

(3) ℙ(MTCθ=Cs|nij,yij)=∏i=1I∏j=1J1−pij(θ|nij,yij)Cs[i,j]pij(θ|nij,yij)1−Cs[i,j]∑s=1(I+JI)∏i=1I∏j=1J1−pij(θ|nij,yij)Cs[i,j]pij(θ|nij,yij)1−Cs[i,j]

where [i,j] represents the entry in the ith row and jth column of the binary matrix $C_{s}$. The contour maximizing Equation (3) is most likely to be the $MTC_{\theta }$ given the current data.

The decision to insert dose levels is made only if ${\text{argmax}}_{C_{s}\in \vartheta }\mathbb{P}(MTC_{\theta }=C_{s}|n_{ij},y_{ij})>\lambda$, for some threshold λ∈[0,1], also referred to as the MTC threshold. Alternative rules to assess the sensitivity of the insertion rule were considered, as outlined in the Supporting Informations.

Note that the idea of studying the probability of contours from the PIPE design is widely applicable to any design, and the uncertainty around the contour has been quantified [28].

### 2.3 Practical Consideration of an Insertion

Once an insertion is triggered, the method must next choose which doses to insert. For simplicity, we assume that discrete dose levels of each agent are available only at the midpoint between each existing dose. While this restriction may align with practice for some medicinal products, we acknowledge that continuous doses may be plausible in other circumstances. Yet with so few patients tested at each combination, estimating an optimal continuous dose to insert carries high uncertainty, so there is limited justification for this approach, even if logistically and commercially viable. An underlying model‐based design could select any continuous dose level in practice, although the model may struggle to handle the jump in toxicity effectively. This consideration is beyond the scope of this paper.

We further assume that extrapolating dose levels outside the initial limits for each agent is forbidden. This aligns with the thinking in practice, where extrapolating dose levels is considered a high‐risk strategy with regard to patient safety.

Insertions are prohibited during the early stages of the trial while limited data is available to support decision‐making. Similarly, insertions are prohibited when the number of patients treated is close to the maximum sample size. The rationale is that there needs to be an adequate sample size remaining to explore at least one of the new combinations to an extent such that it can be recommended for phase II.

The current estimate of the $MTC_{\theta }$ is the driver for which dose level(s) to insert. If part of the $MTC_{\theta }$ separates two existing doses, then the midpoint between these two existing doses is chosen as a new dose to insert. One or more doses for each agent obeying this rule can be inserted simultaneously. A maximum of I+J−2 dose levels can be inserted at any point in the trial. To decide which combination subsequent cohorts receive, the underlying dose‐escalation design chooses from combinations in the expanded grid, ensuring the combination administered directly after the insertion is comprised of at least one of the new dose levels. The remainder of the trial follows the underlying dose‐escalation design. Table 16, [Boxed-text sim70417-fea-0001]provides a useful example of how an insertion alters the combination grid when our method is applied to a case study.

### 2.4 Examples

Suppose there is a trial, with a 3×3 combination space, and target toxicity θ=0.30. Below is R code which describes how we calculate each $p_{ij}(\theta |n_{ij},y_{ij})$ from Equation (2), for priors with hyperparameters a and b.

Listing 1

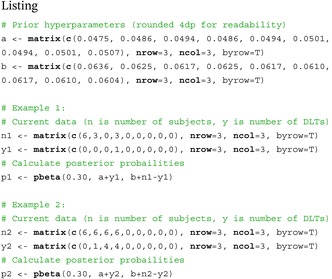

In Example 1, we calculate the posterior probability of each $\pi_{ij}$ being less than or equal to 0.30. Next, we calculate the probability of each of the 20 eligible contours being the true $MTC_{\theta }$, estimated via Equation (3). The highest probability for a given contour is 0.09. We repeat the process in Example 2, after more patients have been recruited. This time, the highest probability for a given contour is 0.68. Assuming 0.68 is greater than our MTC threshold λ, an insertion is triggered. Table 2 illustrates the dose combination space and trial data before and after the insertion in Example 2. The midpoint between the two existing doses is chosen as the new dose to insert.

**TABLE 2:** Dose combination space and trial data before and after the insertion in Example 2. Each entry represents the number of patients experiencing a DLT/number of patients. Inserted dose levels are highlighted in bold. Candidates for the subsequent cohort are denoted by the “+” symbol.

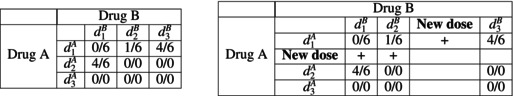

### 2.5 Alternative Proposals

During our research, we considered several methods to address the gap in insertion methods in the combination space. While our main proposal is driven by contours, we also considered two other insertion methods as candidates.

#### 2.5.1 Alternative Proposal 1

This method extends the idea of the single‐agent method proposed by Chu et al. [24]. For each combination, the toxicity probability space is divided into three distinct regions: an underdosing region $R_{1}=[0,0.16)$, an acceptable region $R_{2}=[0.16,0.33]$, and an overdosing region $R_{3}=(0.33,1]$. After each cohort of patients is observed, the quantities $\mathbb{P}(\pi_{ij}\in R_{1}|n_{ij},y_{ij},a_{ij},b_{ij})$ and $\mathbb{P}(\pi_{ij}\in R_{3}|n_{ij},y_{ij},a_{ij},b_{ij})$ are calculated for all combinations, using beta posterior distributions as below. For convenience, these are called $\mathbb{P}(\pi_{ij}\in R_{1})$ and $\mathbb{P}(\pi_{ij}\in R_{3})$ from hereon.

$$\begin{array}{ll} \mathbb{P}(\pi_{ij}\in R_{1}) & =\text{Beta}(0.16,n_{ij}+a_{ij},n_{ij}-y_{ij}+b_{ij})\\ \mathbb{P}(\pi_{ij}\in R_{3}) & =1-\text{Beta}(0.33,n_{ij}+a_{ij},n_{ij}-y_{ij}+b_{ij}). \end{array}$$

To allow fair comparison, priors for a and b are equal to the values used in the main proposal. To ensure toxicity monotonically increases with dose, we ensure $\mathbb{P}(\pi_{1j}\in R_{1})\geq \ldots \geq \mathbb{P}(\pi_{Ij}\in R_{1})\;\forall j$ and $\mathbb{P}(\pi_{i1}\in R_{1})\geq \ldots \geq \mathbb{P}(\pi_{iJ}\in R_{1})\;\forall i$ by assigning each combination the value of its neighbor, should either inequality be in violation. Analogously, we ensure $\mathbb{P}(\pi_{1j}\in R_{3})\leq \ldots \leq \mathbb{P}(\pi_{Ij}\in R_{3})\;\forall j$ and $\mathbb{P}(\pi_{i1}\in R_{3})\leq \ldots \leq \mathbb{P}(\pi_{iJ}\in R_{3})\;\forall i$ by assigning each combination the value of its neighbor.

An insertion is triggered if, for all combinations, the following two conditions are satisfied:

(4) $$\begin{array}{ll} \mathbb{P}(\pi_{ij}\in R_{1})> & C_{1} \end{array}$$

(5) $$\begin{array}{ll} \mathbb{P}(\pi_{ij}\in R_{3})> & C_{2} \end{array}$$

for some cutoff values $C_{1},C_{2}\in [0,1].$ In line with the original paper, we apply the constraint $C_{1}=C_{2}$, such that insertion decisions weight underdosing and overdosing equally.

As in Section 2.3, we assume that discrete dose levels of each agent are available only at the midpoint between each existing dose, and that extrapolating dose levels outside the initial limits for each agent is forbidden. Once an insertion is triggered, insertions occur at the midpoint between $d_{i}$ and $d_{i+1}$ should $\mathbb{P}(\pi_{ij}\in R_{1})>C_{1}$ and $\mathbb{P}(\pi_{i+1j}\in R_{3})>C_{2}$ hold for some i=1,…,I−1. Similarly, insertions occur at the midpoint between $d_{j}$ and $d_{j+1}$ should $\mathbb{P}(\pi_{ij}\in R_{1})>C_{1}$ and $\mathbb{P}(\pi_{ij+1}\in R_{3})>C_{2}$ hold for some j=1,…,J−1. One or more doses for each agent obeying this rule can be inserted simultaneously.

#### 2.5.2 Alternative Proposal 2

This method also extends the idea of the single‐agent method proposed by Chu et al. [24]. As with Alternative Method 1, the toxicity probability space is divided into three distinct regions for each combination: an underdosing region $R_{1}=[0,0.16)$, an acceptable region $R_{2}=[0.16,0.33]$ and an overdosing region $R_{3}=(0.33,1]$. After each cohort of patients is observed, the quantities $\mathbb{P}(\pi_{ij}\in R_{1})$ and $\mathbb{P}(\pi_{ij}\in R_{3})$ are calculated for all combinations; the approach as in Alternative Method 1.

To allow fair comparison, priors for a and b and the approach to ensure monotonicity are the same as applied in Alternative Method 1.

An insertion is triggered if, for some combination $d_{ij}$, the following three conditions are satisfied:

- $\mathbb{P}(\pi_{ij}\in R_{1})>C_{1}$
- $\mathbb{P}(\pi_{i+1j}\in R_{3})>C_{2}$ or $\mathbb{P}(\pi_{ij+1}\in R_{3})>C_{2}$
- The ratio of the number of DLTs to the number of subjects on any combination in the grid is not in the range [1/6, 1/3]. This is to prevent insertions from happening in the presence of existing combinations that a safety committee in practice would prefer to explore.

Cutoff values are such that $C_{1},C_{2}\in [0,1].$ In line with the original paper, we apply the constraint $C_{1}=C_{2}$, such that insertion decisions weight underdosing and overdosing equally. The rules for insertion locations are the same as in Alternative Proposal 1. Note that the more aggressive insertion strategy is one key difference in behavior compared to Alternative Proposal 1.

## 3 Considered Dose‐Escalation Designs

The underlying dose‐escalation designs chosen for this research are the PIPE design (a model‐free design) [27] and the two‐dimensional BLRM (a model‐based design) [15], both of which are well‐established approaches in the combination setting. The remainder of this section reviews the methodology for these two designs.

### 3.1 PIPE Design

As in Section 2.2, the PIPE design models each $\pi_{ij}$ independently, assigning each a beta prior $\pi_{ij}∼\text{Beta}(a_{ij},b_{ij})$ for hyperparameters $a_{ij}$ and $b_{ij}$ ∀i,j. Priors can be prespecified if knowledge of the toxicity of combinations is available. Alternatively, hyperparameters yielding noninformative priors can be chosen in the absence of prior knowledge on the dose‐toxicity relationship. Assuming each patient is independent such that $y_{ij}∼\text{Binomial}(n_{ij},\pi_{ij})$ ∀i,j, the posterior distribution for $\pi_{ij}$ can be written as in Equation (1).

The key idea of the PIPE design is that escalation decisions are driven by the estimate of the $MTC_{\theta }$. Following each cohort, the $MTC_{\theta }$ is re‐estimated based on the current posterior distributions for the $\pi_{ij}$s. Specifically, the contour maximizing Equation (3) is the $MTC_{\theta }$. From admissible combinations adjacent to the contour, one combination is selected for the next cohort based on a weighted randomization procedure. This involves weighting each combination by the inverse of its sample size, with the rationale being varied experimentation around the $MTC_{\theta }$. Admissible combinations are those at most one dose level of either drug away from any previous combination (excluding diagonal escalation), and those not considered too toxic by the overdosing criterion, which is discussed more below.

Escalation continues in this way until all patients are treated, at which point all combinations (which have been experimented on with at least six patients) closest to below the $MTC_{\theta }$ are recommended for phase II. To allow for comparisons within our simulation study, the design is modified to select a single combination for phase II. If multiple combinations are recommended, the one with posterior mean probability of toxicity closest to θ is selected, choosing one at random in the event of a tie.

To limit experimentation on toxic combinations, the design deploys an overdosing rule which considers the expected probability of $d_{ij}$ being above the current $MTC_{\theta }$ estimate averaged over all monotonic contours. This is written as

$$q_{ij}=\underset{C_{s}\in \vartheta }{\sum }\{C_{s}\times \mathbb{P}(MTC_{\theta }=C_{s}|n_{ij},y_{ij})\}$$

and $d_{ij}$ cannot be administered to the next cohort if $q_{ij}\geq \epsilon$ for some ϵ>0. A trial is terminated if combination $d_{11}$ satisfies this condition.

### 3.2 BLRM

The two‐dimensional BLRM uses a five‐parameter function to describe the joint toxicity probability $\pi_{ij}$ for i=1,…,I and j=1,…,J. The design also incorporates the escalation with overdose control (EWOC) principle, which discourages the model from escalating to toxic combinations.

The toxicity probability at $d_{i}^{A}$, $\psi (d_{i}^{A},\alpha_{1},\alpha_{2})$, is defined as

$$\text{logit}(\psi (d_{i}^{A},\alpha_{1},\alpha_{2}))=\alpha_{1}+\alpha_{2}{\tilde{d}}_{i}^{A}.$$

Similarly, the toxicity probability at $d_{j}^{B}$, $\psi (d_{j}^{B},\beta_{1},\beta_{2})$, is defined as

$$\text{logit}(\psi (d_{j}^{B},\beta_{1},\beta_{2}))=\beta_{1}+\beta_{2}{\tilde{d}}_{j}^{B}.$$

The dose levels ${\tilde{d}}_{i}^{A}$ and ${\tilde{d}}_{j}^{B}$ are standardized doses calculated so that the prior estimates of toxicity probabilities are compatible. These prior estimates are assigned prospectively.

If there is no interaction between the two agents, the probability of no toxicity on the combination would factorize to the product of no toxicity on any single agent, so that

$$\psi {\left(d_{ij},\alpha_{1},\alpha_{2},\beta_{1},\beta_{2}\right)}^{†}=1-(1-\psi (d_{i}^{A},\alpha_{1},\alpha_{2}))(1-\psi (d_{j}^{B},\beta_{1},\beta_{2}))$$

where the superscript † stands for no interaction. Then we introduce an interaction parameter, η, to model the interaction between the two agents. This forms part of a term that has the interpretation of an odds multiplier, such that

(6) $${\text{odds}}_{ij}={\text{odds}}_{ij}^{†}\times \exp(\eta \log(1+\exp({\tilde{d}}_{i}^{A})\exp({\tilde{d}}_{j}^{B})))$$

where ${\text{odds}}_{ij}=\psi (d_{ij},\alpha_{1},\alpha_{2},\beta_{1},\beta_{2})/(1-\psi (d_{ij},\alpha_{1},\alpha_{2},\beta_{1},\beta_{2})).$

Therefore, the BLRM has a total of five parameters: $\theta =(\alpha_{1},\alpha_{2},\beta_{1},\beta_{2},\eta )$. Under the Bayesian approach, each of these are assigned normal prior distributions.

$$\begin{array}{ll} \left(\begin{array}{c} \alpha_{1}\\ \log(\alpha_{2}) \end{array}\right) & ∼{\text{MVN}}_{2}\left(\left(\begin{array}{c} \mu_{\alpha_{1}}\\ \mu_{\alpha_{2}} \end{array}\right),\left(\begin{array}{cc} \sigma_{\alpha_{1}}^{2} & 0\\ 0 & \sigma_{\alpha_{2}}^{2} \end{array}\right)\right)\\ \left(\begin{array}{c} \beta_{1}\\ \log(\beta_{2}) \end{array}\right) & ∼{\text{MVN}}_{2}\left(\left(\begin{array}{c} \mu_{\beta_{1}}\\ \mu_{\beta_{2}} \end{array}\right),\left(\begin{array}{cc} \sigma_{\beta_{1}}^{2} & 0\\ 0 & \sigma_{\beta_{2}}^{2} \end{array}\right)\right)\\ \eta & ∼N(\mu_{\eta },\sigma_{\eta }^{2}) \end{array}$$

The likelihood is a product of Bernoulli densities proportional to $\prod_{i=1}^{I}\prod_{j=1}^{J}\pi_{ij}^{y_{ij}}{\left(1-\pi_{ij}\right)}^{n_{ij}-y_{ij}}$. After each cohort is observed, samples of each parameter are drawn from their full conditional posterior distributions using MCMC methods. The posterior mean of each parameter is estimated and substituted into Equation (6). This yields an estimate for each posterior mean probability of toxicity to guide the escalation process.

The combination (also obeying the neighboring constraint and EWOC principle described below) with posterior mean probability of toxicity closest to θ from below is administered to the next cohort. This is also referred to as a patient gain approach, as it prioritizes patient safety over exploring previously untested combinations [29]. If no combinations satisfy the constraints, the trial is terminated. The trial continues until the sample size has been exhausted, and the design selects the combination with posterior mean probability of toxicity closest to θ from below, provided at least six patients have been treated on that combination.

During the escalation phase, the BLRM can only escalate to combinations satisfying the neighborhood constraint and EWOC principle. The neighborhood constraint prevents escalation or de‐escalation to any combination that is more than one dose level of either drug away from any previous combination, and also prevents diagonal escalation. The EWOC principle states that $d_{ij}$ can only be administered if $\mathbb{P}(\pi_{ij}>\zeta )\leq \epsilon$ for some threshold ϵ>0, and ζ∈(0,1].

## 4 Simulation Study

### 4.1 Setting

To assess the performance of our insertion procedure, we conduct a comprehensive simulation study across each of the 12 scenarios in Table 3. The objective of each trial is to identify the maximum tolerated dose combination, with a target toxicity of θ=0.30. Trials consist of Agents A and B. Agent A has three doses $d_{1}^{A}$, $d_{2}^{A}$ and $d_{3}^{A}$, as does Agent B with $d_{1}^{B}$, $d_{2}^{B}$ and $d_{3}^{B}$. The starting combination is $d_{11}$. Patients are recruited in cohorts of three, with a maximum sample size of 48, chosen to be logistically plausible while enabling adequate exploration of potential new combinations inserted mid‐trial. The insertion procedure is applied to trials using two underlying dose‐escalation designs: the PIPE design and the BLRM.

**TABLE 3:** Toxicity scenarios to assess model performance in the simulation study. Rows and columns refer to the dose levels of Agent A and B, respectively. “**Correct**” combinations are in bold. “Acceptable” combinations are underlined. “Toxic” combinations are red.

	$d_{1}^{B}$	$d_{2}^{B}$	$d_{3}^{B}$		$d_{1}^{B}$	$d_{2}^{B}$	$d_{3}^{B}$		$d_{1}^{B}$	$d_{2}^{B}$	$d_{3}^{B}$
Scenario A1				Scenario A2				Scenario A3			
$d_{1}^{A}$	0.10	0.45	0.50	$d_{1}^{A}$	0.05	0.10	0.45	$d_{1}^{A}$	0.05	0.05	0.10
$d_{2}^{A}$	0.45	0.50	0.60	$d_{2}^{A}\;$	0.10	0.45	0.50	$d_{2}^{A}$	0.05	0.10	0.45
$d_{3}^{A}$	0.50	0.60	0.65	$d_{3}^{A}$	0.45	0.50	0.60	$d_{3}^{A}$	0.10	0.45	0.50

Scenario B1				Scenario B2				Scenario B3			
$d_{1}^{A}$	0.20	0.45	0.50	$d_{1}^{A}$	0.05	0.20	0.45	$d_{1}^{A}$	0.05	0.10	0.20
$d_{2}^{A}$	0.45	0.50	0.60	$d_{2}^{A}$	0.20	0.45	0.50	$d_{2}^{A}$	0.10	0.20	0.45
$d_{3}^{A}$	0.50	0.60	0.65	$d_{3}^{A}$	0.45	0.50	0.60	$d_{3}^{A}$	0.20	0.45	0.50

Scenario C1				Scenario C2				Scenario C3			
$d_{1}^{A}$	0.10	**0.30**	0.45	$d_{1}^{A}$	0.05	0.10	**0.30**	$d_{1}^{A}$	0.05	0.05	0.10
$d_{2}^{A}$	**0.30**	0.45	0.50	$d_{2}^{A}$	0.10	**0.30**	0.45	$d_{2}^{A}$	0.05	0.10	**0.30**
$d_{3}^{A}$	0.45	0.50	0.60	$d_{3}^{A}$	**0.30**	0.45	0.50	$d_{3}^{A}$	0.10	**0.30**	0.45

Scenario D1				Scenario D2				Scenario D3			
$d_{1}^{A}$	0.20	**0.30**	0.45	$d_{1}^{A}$	0.05	0.20	**0.30**	$d_{1}^{A}$	0.05	0.05	0.20
$d_{2}^{A}$	**0.30**	0.45	0.50	$d_{2}^{A}$	0.20	**0.30**	0.45	$d_{2}^{A}$	0.05	0.20	**0.30**
$d_{3}^{A}$	0.45	0.50	0.60	$d_{3}^{A}$	**0.30**	0.45	0.50	$d_{3}^{A}$	0.20	**0.30**	0.45

Combinations with true probability of toxicity in the ranges [0.275, 0.325] and [0.16, 0.33] are labelled correct and acceptable, respectively. Combinations with true probability of toxicity in the ranges [0, 0.16) and (0.33, 1] are labeled subtherapeutic and toxic, respectively.

The scenarios in Table 3 have been selected, differing in which part of the grid includes combinations close to θ, if any. More specifically, Scenarios A1, A2, and A3 contain no acceptable combinations. Scenarios B1, B2, and B3 contain at least one acceptable combination, yet no correct combinations. Scenarios C1, C2, and C3 contain multiple correct combinations, yet lack other combinations with acceptable toxicity to explore. Scenarios D1, D2, and D3 contain multiple correct combinations, in addition to more combinations at other acceptable toxicity levels to explore.

To fully explore the behavior of our insertion procedure, we run 1000 simulations for each scenario for different λ thresholds, namely λ=0.5,0.6,…,1. A threshold of λ=1 is equivalent to turning off the insertion procedure, providing a useful reference case. Lower values of λ are consistent with the rules for dose insertions being more lenient. In the mathematical sense, decreasing λ reduces the certainty needed in identifying one contour as the $MTC_{\theta }$ before making an insertion. Note we only explore values of λ≥0.5 to prevent insertions occurring where two contours are approximately equally as probable to be the $MTC_{\theta }$ (due to at least one $\pi_{ij}$ being close to θ). In this case, treating more patients at $d_{ij}$ is preferable to inserting new dose levels.

Insertions are prohibited until at least 18 patients have been treated in total, and if more than 42 patients have been treated in total. For simplicity, insertions are restricted to occur at a maximum of one timepoint per trial. The hyperparameters of the beta prior distributions within the insertion framework are chosen to yield noninformative priors due to the absence of prior knowledge. For consistency, the prior means and sample sizes are the same as those chosen in Section 4.3.

For computational reasons, the BLRM uses an MCMC approach to draw 2000 samples for each parameter, before the posteriors are estimated. The EWOC principle is applied such that $d_{ij}$ can only be administered if $\mathbb{P}(\pi_{ij}>0.33)\leq \epsilon$ for some threshold ϵ>0.

Lastly, we comment on the true probability of toxic response assigned to dose levels, should they be added mid‐trial in our simulation study. These probabilities are interpolated based on the probabilities at adjacent combinations, ensuring the monotonicity assumption is obeyed.

### 4.2 Performance Metrics

In this section, we introduce all the performance metrics used in our results. Firstly, the proportion of trials in which at least one dose insertion occurred will be presented for each scenario. The following operating characteristics will also be presented for each scenario: the proportion of correct selections (PCS); the proportion of acceptable selections (PAS); the proportion of subtherapeutic selections (PSS); and the proportion of toxic selections (PTS). Combinations with true probability of toxicity in the ranges [0.275, 0.325] and [0.16, 0.33] are labeled correct and acceptable, respectively. Combinations with true probability of toxicity in the ranges [0, 0.16) and (0.33, 1] are labeled subtherapeutic and toxic, respectively.

The Accuracy Index, as defined in Equation (7), will be presented to measure the quality of selections. This is achieved by calculating the differences between the true toxicity of each selected combination and the target θ. Note $\rho_{ij}$ is the proportion of trials selecting combination $d_{ij}$. Higher values of the Accuracy Index are associated with higher quality selections.

(7) $$\text{Accuracy}\text{Index (\%)}=100-\left[100\times I\times J\times \frac{\sum_{i=1}^{I}\sum_{j=1}^{J}{\left(\pi_{ij}-\theta \right)}^{2}\times \rho_{ij}}{\sum_{i=1}^{I}\sum_{j=1}^{J}{\left(\pi_{ij}-\theta \right)}^{2}}\right]$$

The Utility Index, as defined in Equation (8), will be presented as an alternative way of measuring the quality of selections. This is achieved by rewarding correct and acceptable selections and penalizing toxic selections. Higher values of the Utility Index are associated with higher quality selections.

(8) Utility Index (%)=PCS+PAS−2×PTS

Finally, to assess the effectiveness and safety of the dose‐finding procedure, we will present the average number of patients treated on acceptable and toxic combinations for selected values of λ.

### 4.3 Prior Specification

The hyperparameters specified in the prior distributions of all model‐based and model‐free designs have a considerable impact on their behavior. Since our simulation study aims to assess the performance of our insertion procedure applied to the PIPE design and BLRM across 12 scenarios, the fairest approach is to specify “operational priors” for each design. These are prior distributions tuned to consistently make recommendations close to the target toxicity, and that limit the risk of toxic recommendations, for all scenarios.

For the PIPE design, we use the two‐stage calibration approach from Barnett et al. [10] to specify prior distributions for the probability of toxicity at each combination and the overdosing threshold. The first stage involves fixing the overdosing threshold and employing a grid search over values used to construct the operational priors. Explicitly, these are the probability of toxic response at the lowest combination, the magnitude of the increments in probability of toxic response between adjacent combinations (these two enable calculation of the prior mean at each combination), and the prior sample size (which is constrained to be equal at each combination). The second stage involves fixing the optimal prior means and sample sizes from the first stage and employing a search over a set of feasible overdosing threshold values.

The prior means and sample sizes were selected according to the results from Barnett et al. [10]. The prior means, as pictured in Table 4, are replicates of those chosen in the reference paper. The prior means for newly inserted combinations were interpolated using the mean of adjacent combinations. Each combination was assigned a prior sample size of 1/(I×J). This is so that it varies from 1/9 at the start of the trial, and decreases to as small as 1/25, in the instance the combination space increases to its maximum size of 5×5 following insertions. This ensures the prior sample sizes are always close to the optimal value 1/18 chosen in the reference paper. The threshold for the overdosing rule, ϵ=0.5, was also chosen based on the reference paper.

**TABLE 4:** PIPE design prior means for each starting combination in the 3×3 combination space. Note each starting combination was assigned a prior sample size of 1/9.

	$d_{1}^{B}$	$d_{2}^{B}$	$d_{3}^{B}$
$d_{1}^{A}$	0.05	0.075	0.10
$d_{2}^{A}$	0.075	0.10	0.125
$d_{3}^{A}$	0.10	0.125	0.15

Given the calibrated prior means and sample sizes, an optimization algorithm was then used to generate the corresponding prior hyperparameters at each combination [27].

For the BLRM, we used a cyclic calibration approach to define the set of operational priors, the prior toxicity probabilities for each agent, which allow for calculation of standardized doses, and the EWOC threshold. The calibration approach is similar to that of Pavel et al. [30]. Namely, the hyperparameters in this approach were: mean and variance of the intercept parameters; mean and variance of the slope parameters; variance of the interaction parameter; prior toxicity probabilities assigned to the lowest dose for each agent; the fixed increase in the prior toxicity probabilities assigned to adjacent dose levels for each agent; and the EWOC threshold.

Scenarios Z1 and Z2 in Table 5 were chosen to perform the cyclic calibration approach. Chen et al. [31] established that calibration on a small subset of scenarios (covering the hardest and simplest cases) led to choosing operational priors with similar operating characteristics when compared to using all scenarios, while also greatly reducing computational expense. For each combination of hyperparameters in both scenarios, PCS and PTS were measured from 500 simulations. The following predetermined decision rule was used to choose the set of hyperparameters for the operational priors: choose the set of hyperparameters maximizing the geometric mean PCS, while constraining the PTS to at most 25% in Scenario Z1.

**TABLE 5:** Toxicity scenarios to assess BLRM performance in the calibration study. Rows and columns refer to the dose levels of Agent A and B, respectively. “**Correct**” combinations are in bold. “Acceptable” combinations are underlined. “Toxic” combinations are red.

	Scenario Z1				Scenario Z2		
	$d_{1}^{B}$	$d_{2}^{B}$	$d_{3}^{B}$		$d_{1}^{B}$	$d_{2}^{B}$	$d_{3}^{B}$
$d_{1}^{A}$	**0.30**	0.40	0.50	$d_{1}^{A}$	0.10	0.20	0.25
$d_{2}^{A}$	0.40	0.50	0.65	$d_{2}^{A}$	0.20	0.25	**0.30**
$d_{3}^{A}$	0.50	0.60	0.70	$d_{3}^{A}$	0.40	0.50	0.60

The predetermined decision rule resulted in the following operational priors being chosen from the cyclic calibration approach.

$$\begin{array}{ll} \left(\begin{array}{c} \alpha_{1}\\ \log(\alpha_{2}) \end{array}\right) & ∼{\text{MVN}}_{2}\left(\left(\begin{array}{c} -\;2\\ 0.5 \end{array}\right),\left(\begin{array}{cc} 1 & 0\\ 0 & 0.25 \end{array}\right)\right)\\ \left(\begin{array}{c} \beta_{1}\\ \log(\beta_{2}) \end{array}\right) & ∼{\text{MVN}}_{2}\left(\left(\begin{array}{c} -\;2\\ 0.5 \end{array}\right),\left(\begin{array}{cc} 1 & 0\\ 0 & 0.25 \end{array}\right)\right)\\ \eta & ∼N(0,0.25) \end{array}$$

The prior toxicity probabilities chosen for both agents from the cyclic calibration approach were (0.10, 0.25, 0.40). This meant the standardized doses could be calculated subject to the condition $\left({\tilde{d}}_{1}^{A},{\tilde{d}}_{2}^{A},{\tilde{d}}_{3}^{A})=({\tilde{d}}_{1}^{B},{\tilde{d}}_{2}^{B},{\tilde{d}}_{3}^{B})=(0.10,0.25,0.40\right)$. Lastly, the EWOC threshold was chosen as ϵ=0.40. For newly inserted combinations, prior toxicity probabilities were interpolated using the mean of adjacent doses. Operational priors and the EWOC threshold were not changed.

### 4.4 Results: PIPE Design

**FIGURE 1:**
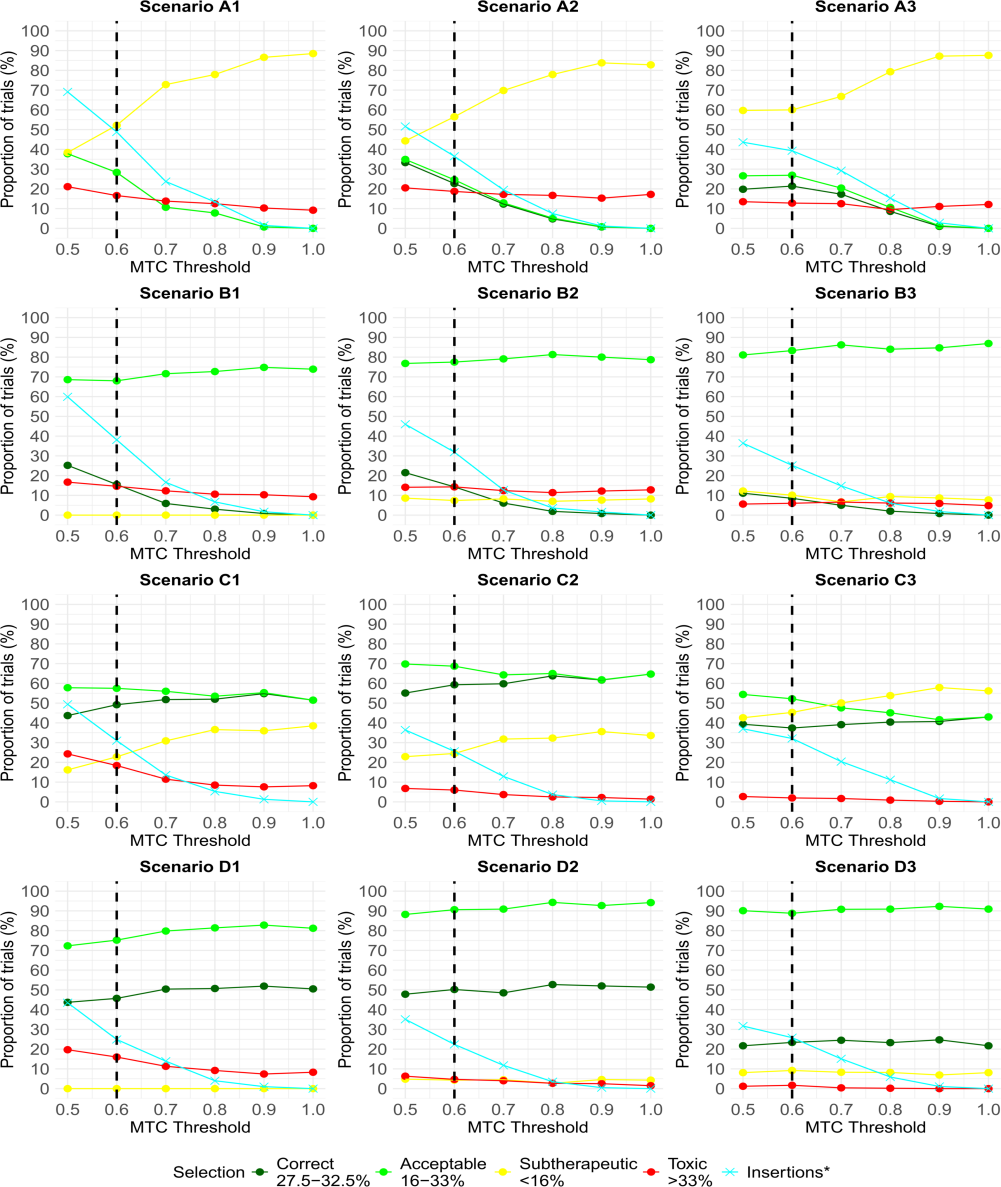
Plots of the operating characteristics at each scenario for the PIPE design at different MTC thresholds (or λ). An MTC threshold of 1 is equivalent to the insertion procedure being turned off. * refers to the proportion of trials where an insertion was made. The dashed line at λ=0.6 corresponds to the threshold recommended by the authors.

Figure 1 presents the operating characteristics for each of the 12 scenarios for the PIPE design at different MTC thresholds, namely λ=0.5,0.6,…,1. A threshold of λ=1 is equivalent to turning off the insertion procedure. In all cases, decreasing λ leads to a greater proportion of trials having dose insertions, shown by the cyan line.

Scenarios A1, A2, and A3 (which contain no acceptable combinations in the initial grid) follow a similar trend. Decreasing λ coincides with large increases in the PCS and PAS. Notably, the PAS increases from 0% to as much as 37.8%, 34.9%, and 26.6%, respectively, when λ decreases from 1 to 0.5, demonstrating merit in the insertion procedure. There is no increase in the PTS in A2 and A3, and only a minor increase in A1, largely due to selections with a probability of toxicity just above 33%. We observe substantial decreases in the PSS, from above 80% in all three scenarios when insertions are prohibited, to between 40% and 60% when λ=0.5.

We observe a benefit when decreasing λ in Scenarios B1, B2, and B3 (which contain no correct combinations). In B1 and B2 in particular, approximately half of the trials with an insertion recommend a correct combination. The PCS increases from 0% to 25.2% and 21.5% respectively when λ decreases from 1 to 0.5. Importantly, there is no meaningful change in the PAS, PTS, and PSS.

One could argue that inserting dose levels into Scenarios C1, C2, and C3 is beneficial for more exploration at acceptable combinations. In all three scenarios, a trade‐off in the PAS and PCS is observed when decreasing λ from 1 to 0.5. The PAS increases slightly, while the PCS decreases slightly. An advantage of insertions is that the PTS and PSS are mostly unaffected.

Scenarios D1, D2, and D3 represent cases where insertions are unnecessary since target combinations are already in the original grid. Despite the number of insertions increasing to over 30% in Scenarios D2 and D3 when λ decreases from 1 to 0.5, all operating characteristics remain consistent. In D1, we observe a small drop‐off in the PCS and PAS at lower λ values. Across these three scenarios, insertions largely do not lead to poorer selections at the end of the trial. Insertion decisions are data‐driven, and therefore intuitive, despite target combinations belonging to the original grid. Greater values of λ lead to fewer unnecessary insertions in these scenarios. However, the tuning of λ must consider its impact on operating characteristics across a wide range of scenarios, since the landscape is unknown before a trial begins.

In summary, when applied to the PIPE design, the insertion procedure can substantially increase the probability of selecting combinations with toxicity close to the target level in scenarios where few or no combinations with acceptable toxicity are present at the beginning of the trial. The probability of toxic selections is mostly unaffected. Moreover, the insertion procedure does not negatively impact performance when inserted combinations do not have toxicity close to the target level.

**FIGURE 2:**
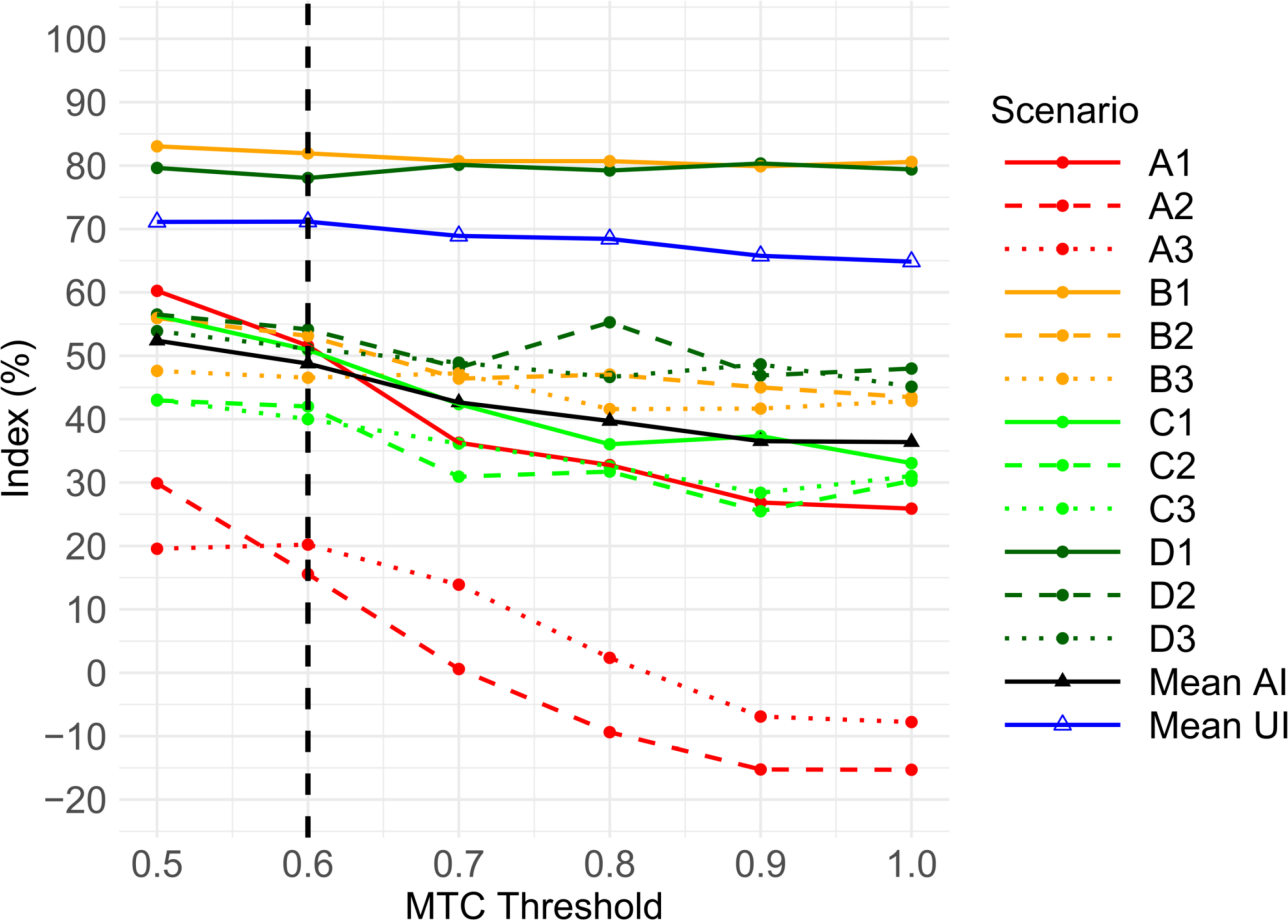
Plot showing the Accuracy Index per scenario, mean Accuracy Index and mean Utility Index, against the MTC threshold (or λ) for the PIPE design. An MTC threshold of 1 is equivalent to the insertion procedure being turned off. The dashed line at λ=0.6 corresponds to the threshold recommended by the authors. AI = Accuracy Index. UI = Utility Index.

Figure 2 shows that the Accuracy Index for the PIPE design increases as λ decreases in all 12 scenarios. The mean Accuracy Index also captures this trend, strictly increasing from 0.364 when λ=1, to 0.524 when λ=0.5. The greatest increases in the Accuracy Index are in Scenarios A1, A2, and A3, where no acceptable combinations exist in the initial dosing grid. Figure 2 also shows that the mean Utility Index tends to increase as λ decreases from 1 to 0.5, peaking slightly at λ=0.6. Based on the available evidence, we recommend using an MTC threshold of λ=0.6 for the PIPE design.

Lastly, applying the insertion procedure has a negligible effect on the toxicity that patients are exposed to during the trial. Table 6 demonstrates this, showing the average number of patients treated on acceptable and toxic combinations for λ=0.6 and λ=1. On average, the insertion procedure leads to 1.1 more patients being treated on acceptable combinations and 0.9 more patients being treated on toxic combinations.

**TABLE 6:** Mean number of patients treated at acceptable and toxic combinations for the PIPE design for MTC thresholds of λ=0.6 and λ=1 (insertions turned off).

Scenario	Mean number of patients treated at acceptable combinations λ=0.6	Mean number of patients treated at acceptable combinations λ=1 (off)	Mean number of patients treated at toxic combinations λ=0.6	Mean number of patients treated at toxic combinations λ=1 (off)
A1	7.6	0.0	23.3	21.8
A2	6.1	0.0	18.8	19.0
A3	5.4	0.0	9.7	9.9
B1	22.5	24.1	21.9	20.2
B2	26.7	26.7	16.1	16.2
B3	32.2	32.7	5.7	5.6
C1	23.5	23.3	16.1	12.8
C2	29.7	28.8	3.8	2.5
C3	17.7	15.8	0.7	0.1
D1	31.4	33.4	14.4	12.6
D2	40.2	41.6	3.5	2.4
D3	37.1	38.6	0.6	0.1
Mean	23.30	22.10	11.20	10.30

### 4.5 Results: BLRM

**FIGURE 3:**
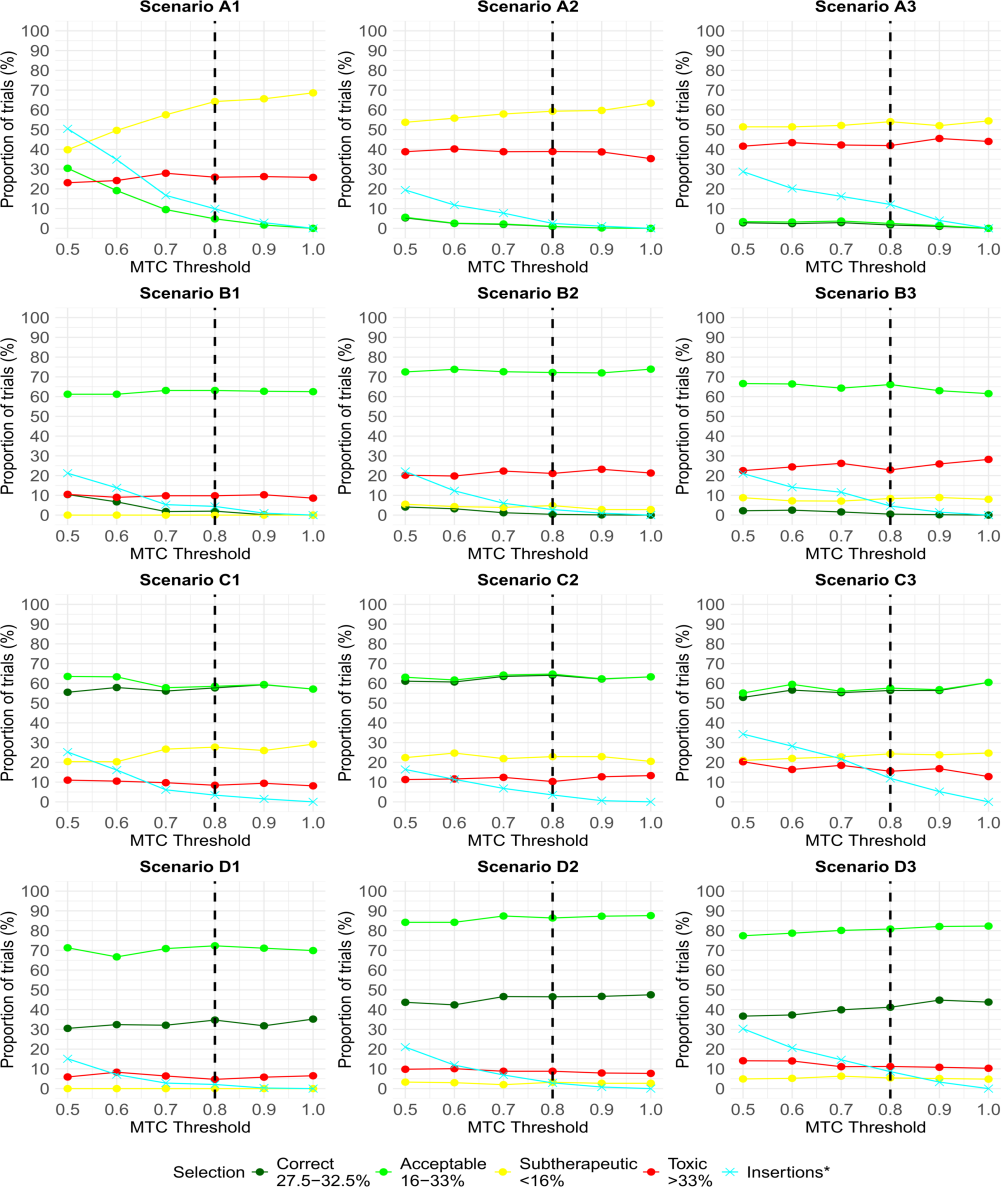
Plots of operating characteristics for the BLRM at different MTC thresholds (or λ). * refers to the proportion of trials where an insertion was made. The dashed line at λ=0.8 corresponds to the threshold recommended by the authors.

Similar to the PIPE design above, Figure 3 presents the operating characteristics for each of the 12 scenarios for the BLRM at different MTC thresholds, namely λ=0.5,0.6,…,1. A threshold of λ=1 is equivalent to turning off the insertion procedure. In all cases, decreasing λ leads to a greater proportion of trials having dose insertions, shown by the cyan line.

Compared to the PIPE design, decreasing λ generally has less impact on the operating characteristics. Two possible reasons for this are as follows. Firstly, the underlying BLRM explores the combination space less effectively, resulting in fewer insertions in scenarios lacking correct or acceptable combinations. Secondly, even after insertions occur, the design does not behave optimally as the parametric function underpinning the model does not handle sharp increments in toxicity effectively.

The PAS increases most steeply in Scenario A1 from 0% to approximately 30% when decreasing λ from 1 to 0.5. Increases in PAS are very shallow in Scenarios A2 and A3. These increases occur with minimal impact on the PTS.

In general, changes in the operating characteristics are most prominent when an insertion is desirable in the lower part of the grid, for example, in Scenarios A1, B1, and C1, where the parametric model estimates the dose‐toxicity relationship well. The insertion procedure has minimal effect on the PAS and PCS in other scenarios. The PTS remains consistent when changing λ across all scenarios.

In Scenarios D1, D2, and D3, where insertions are unnecessary since target combinations are already in the original grid, the proportion of insertions is low. This is also true for thresholds closer to λ=0.5.

**FIGURE 4:**
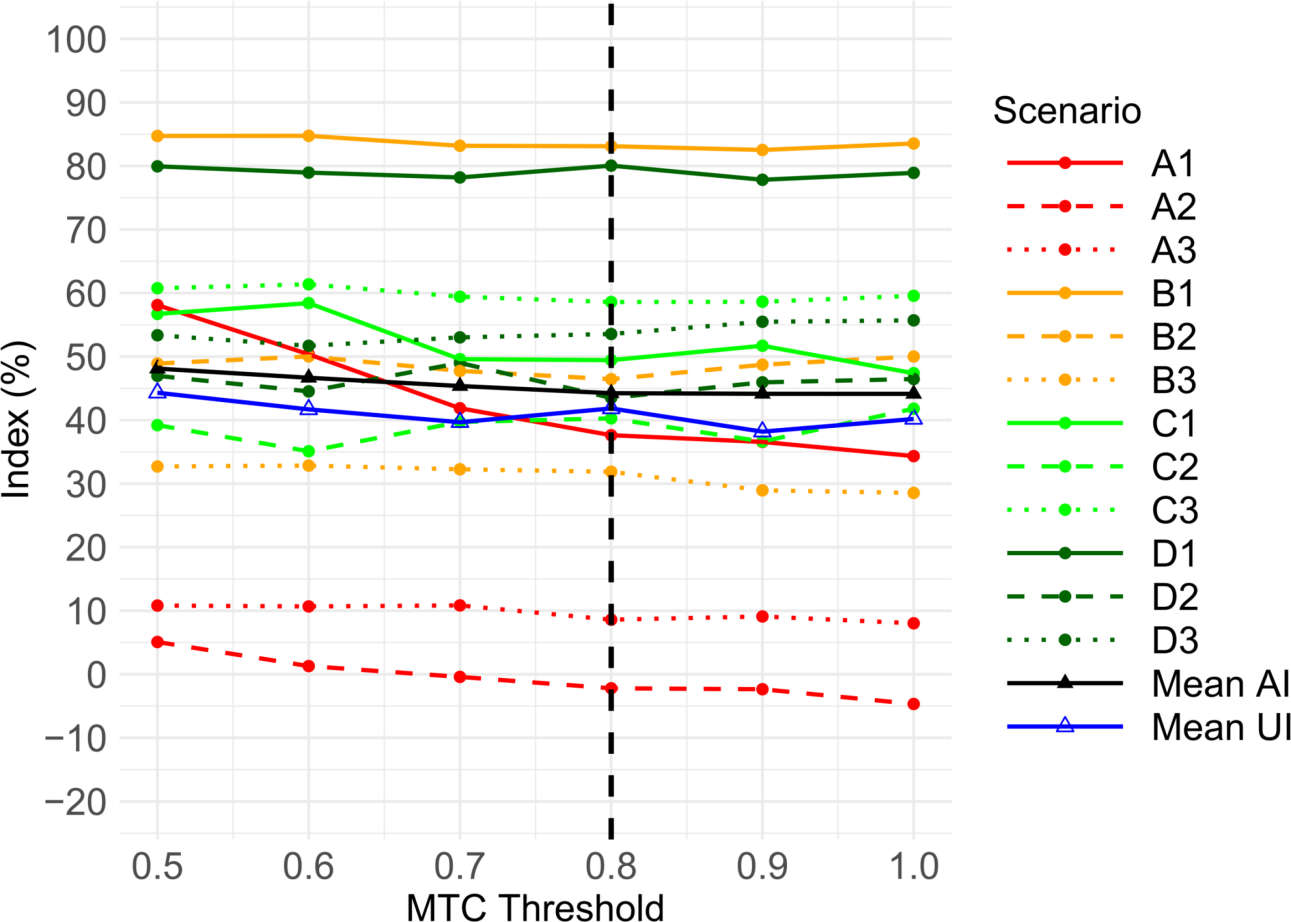
Plot showing the Accuracy Index per scenario, mean Accuracy Index and mean Utility Index, against the MTC threshold (or λ) for the BLRM. An MTC threshold of 1 is equivalent to the insertion procedure being turned off. The dashed line at λ=0.8 corresponds to the threshold recommended by the authors. AI = Accuracy Index. UI = Utility Index.

Figure 4 reveals that the only case showing a marked increase in the Accuracy Index with decreasing λ is Scenario A1. The Accuracy Index is flat in other scenarios, and so is the mean. Figure 4 also lacks a clear trend with respect to the Utility Index. While the peak is at λ=0.5, this can almost entirely be attributed to Scenario A1. By excluding A1 when calculating the mean Utility Index, a λ=0.8 yields the highest result. Based on the available evidence, we recommend using an MTC threshold of λ=0.8 for the BLRM.

As with PIPE design, applying the insertion procedure to the BLRM has a negligible effect on the toxicity that patients are exposed to during the trial. To demonstrate this, we present the average number of patients treated on acceptable and toxic combinations for λ=0.8 and λ=1 shown in Table 7. On average, the insertion procedure leads to 0.1 more patients being treated on acceptable combinations and 0.1 fewer patients being treated on toxic combinations. The minimal difference can in part be attributed to the low proportion of trials with insertions and the inability of the BLRM to explore inserted dose levels in most scenarios.

**TABLE 7:** Mean number of patients treated at acceptable and toxic combinations for the BLRM using MTC thresholds of λ=0.8 and λ=1 (insertions turned off).

Scenario	Mean number of patients treated at acceptable combinations λ=0.8	Mean number of patients treated at acceptable combinations λ=1 (off)	Mean number of patients treated at toxic combinations λ=0.8	Mean number of patients treated at toxic combinations λ=1 (off)
A1	1.2	0.0	17.3	17.9
A2	0.1	0.0	19.8	19.3
A3	0.7	0.0	18.2	18.1
B1	27.5	27.4	10.1	9.9
B2	24.7	25.3	14.0	14.3
B3	21.2	20.7	12.1	12.5
C1	22.8	21.8	7.3	7.7
C2	21.1	21.4	7.6	8.1
C3	17.0	17.2	8.1	7.9
D1	34.5	34.1	4.3	4.9
D2	34.1	35.0	5.8	5.2
D3	28.4	29.2	6.1	5.7
Mean	19.4	19.3	10.9	11.0

#### 4.5.1 Further Considerations

The BLRM is a well‐established model‐based design used for dose‐finding studies; however, there are known disadvantages in both the single‐agent and dual‐agent setting [32]. In this section, we illustrate where it performs poorly and give possible justification. We also draw comparisons between that of the behavior of the PIPE design.

Firstly, the BLRM is prone to model misspecification. We show that its parametrization does not allow for adequate modeling of sharp increases in toxicity, which are plausible in this setting. The simple example trial in Table 8 gives the number of patients with a toxic response out of the number of patients at each combination. Table 9 gives estimates of the posterior mean probability of toxic response (%) generated by the BLRM at each combination (note these are subject to small amounts of random variation depending on the seed). Here, the model greatly overestimates $\pi_{11}$ and underestimates $\pi_{12}$ and $\pi_{21}$, which may lead to poor exploration of the grid and a poor recommendation at the end of the trial.

**TABLE 8:** Example number of patients with a toxic response out of the number of patients. This data was used to generate the estimates shown in Tables 9 and 10.

	$d_{1}^{B}$	$d_{2}^{B}$	$d_{3}^{B}$
$d_{1}^{A}$	0/12	9/12	0
$d_{2}^{A}$	8/12	0	0
$d_{3}^{A}$	0	0	0

**TABLE 9:** BLRM; estimates of the posterior mean probability of toxic response (%).

	$d_{1}^{B}$	$d_{2}^{B}$	$d_{3}^{B}$
$d_{1}^{A}$	24.9	53.6	75.5
$d_{2}^{A}$	47.6	68.8	83.8
$d_{3}^{A}$	67.4	80.9	90.1

In contrast, Table 10 gives estimates of the posterior mean probabilities of toxic response (%) generated by the PIPE design. The noninformative priors allow the data to almost solely drive these estimates. At the three tested combinations, the PIPE design establishes reasonable estimates of the toxicity, which it can use to make effective escalation decisions.

**TABLE 10:** PIPE design; estimates of the posterior mean probability of toxic response (%).

	$d_{1}^{B}$	$d_{2}^{B}$	$d_{3}^{B}$
$d_{1}^{A}$	0.4	74.4	45.6
$d_{2}^{A}$	66.5	45.6	46.5
$d_{3}^{A}$	45.6	46.5	47.2

Secondly, the BLRM escalation rule (which uses a patient gain approach) can lead to poor exploration of the dosing grid. The next combination administered depends upon which combination the model believes has toxicity closest to the target level. In our work, we observe plausible untested candidate combinations, typically in the corners of the dosing grid, being ignored by the model. In contrast, the PIPE design uses the inverse sample size to choose among admissible combinations, allowing for varied experimentation around $MTC_{\theta }$ [27].

Due to these two reasons, we discourage input from the working BLRM parametric function when determining when and where to insert doses mid‐trial. We also note that inserting new combinations close to the target toxicity may support the BLRM and allow it to model the dose‐toxicity relationship more accurately.

### 4.6 Results: Alternative Proposals

We test Alternative Proposals 1 and 2 in the same simulation study as our main proposal, as described in Section 4.1, using the PIPE design as the underlying dose‐escalation method. Instead of varying λ, we vary values for $C_{1}=C_{2}=0.5,0.6,\ldots ,1.$ A threshold of $C_{1}=C_{2}=1$ is equivalent to turning off the insertion procedure, and lower cutoff values are consistent with the rules for dose insertions being more lenient. To compare these methods against our main proposal, suitable values for $C_{1}$ and $C_{2}$ are chosen after applying the approach used for choosing λ=0.6 in Section 4.4. This yields $C_{1}=C_{2}=0.7$ for Alternative Proposal 1 and $C_{1}=C_{2}=0.6$ for Alternative Proposal 2.

**FIGURE 5:**
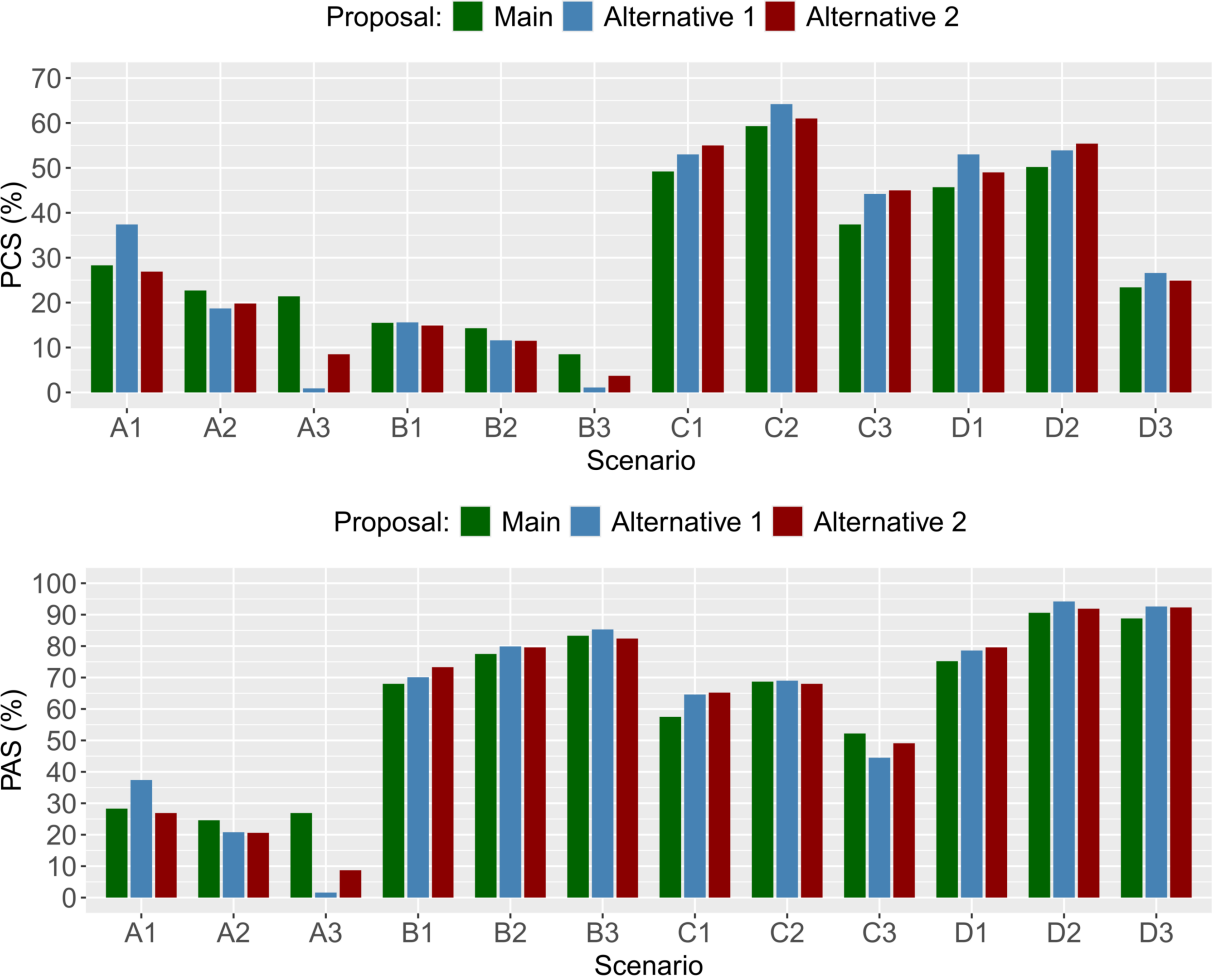
Barplots of the PCS and PAS for the main proposal, Alternative Proposal 1, and Alternative Proposal 2, by scenario. PCS = proportion of correct selections. PAS = proportion of acceptable selections.

Tables 11 and 12 and Figure 5 show some key operating characteristics from our main proposal and Alternative Proposals 1 and 2, using the cutoff values stated above. These can be used to compare each method and their behaviors. In Table 11 we examine Scenarios A1, A2, and A3 closely, where no acceptable combinations are in the starting grid. The alternative proposals find it difficult to identify the need for insertions in Scenario A3, which are necessary between higher dose levels, and therefore have very low PCS and PAS. We believe this more consistent performance makes the main proposal the best candidate for dose insertions. Other advantages include how it deals with monotonicity automatically in the construction of its contours, and that it depends on the target toxicity θ and tuning of λ only, rather than upper and lower toxicity interval bounds and cutoffs $C_{1}$ and $C_{2}$.

**TABLE 11:** The PCS and PAS in Scenarios A1, A2, and A3 for the main proposal, Alternative Proposal 1, and Alternative Proposal 2. PCS = proportion of correct selections. PAS = proportion of acceptable selections.

	Scenario					
	A1		A2		A3	
Method	PCS (%)	PAS (%)	PCS (%)	PAS (%)	PCS (%)	PAS (%)
Main proposal	28.30	28.30	22.70	24.60	21.40	26.90
Alternative proposal 1	37.40	37.40	18.70	20.80	0.90	1.60
Alternative proposal 2	26.90	26.90	19.80	20.60	8.50	8.70

**TABLE 12:** Operating characteristics for the main proposal, Alternative Proposal 1, and Alternative Proposal 2. PCS = proportion of correct selections. PAS = proportion of acceptable selections. PTS = proportion of toxic selections.

Method	PCS (%)		PAS (%)		PTS (%)		Mean insertions (%)	
	Mean	SD	Mean	SD	Mean	SD	A1, A2, A3	D1, D2, D3
Main proposal	31.32	16.54	61.80	24.06	10.98	6.43	41.53	24.37
Alternative proposal 1	31.68	22.13	61.55	29.37	9.57	6.98	31.07	12.87
Alternative proposal 2	31.30	20.58	61.47	28.56	9.34	6.30	34.73	14.40

The proportion of insertions in Scenarios A1, A2, and A3 is highest for the Main Proposal, which leads to higher PCS and PAS. The tradeoffs with the consistent performance in the main proposal are the marginally higher mean PTS, and a higher false insertion rate shown in the last column (since Scenarios D1, D2, and D3 contain multiple combinations at the target toxicity already). Importantly, the higher insertion rate does not result in lower PCS or PAS.

Figure 5 displays a scenario‐by‐scenario comparison of the PCS and PAS for each of the proposals. In particular, these illustrate the superior performance of the main proposal in Scenarios A2 and A3.

## 5 Application to a Trial

The simulation study gives insight into the operating characteristics of the PIPE design and BLRM with the insertion procedure applied. However, for further insight into the escalation behavior, we apply our methods to an example case study. We consider a phase I oncology (breast and lung cancer) study enrolling patients to dosing combinations of four dose levels of neratinib and temsirolimus [33]. A total sample size of 60 patients (cohorts of size 2 or 3) were treated on 12 of 16 possible dosing combinations. Results from 52 patients were included and 10 DLTs were observed, with full results of the trial displayed in Table 13. Note the sharp increase in toxicity observed in the raw trial data, escalating from the (160 mg, 50 mg) combination in either treatment, which appears to lend itself to a dose insertion.

**TABLE 13:** The raw trial data of the study by Gandhi et al. [33]. Each entry represents the number of patients with a DLT/number of patients. The two combinations recommended for phase II from the study are highlighted in bold.

	Temsirolimus				
Neratinib	**Raw trial data**	15 mg	25 mg	50 mg	75 mg
	120 mg	0/2	0/4	1/5	0/4
	160 mg	1/4	1/4	**0/5**	3/6
	200 mg	0/4	**1/8**	1/2	
	240 mg	2/4			

The purpose of this case study is to give an illustration of how the PIPE design (with λ=0.6) and the BLRM (with λ=0.8) explore the dosing grid, given identical patient responses. In order to use the calibrated prior specifications, and in line with the simulation study, we restrict the dosing grid to three doses of each drug, removing the lowest dose of temsirolimus and the highest dose of neratinib. We also fix the cohort size to three patients and the maximum total sample size to 48.

To ensure a fair comparison between designs, we define a fixed set of 48 ordered patient responses for each dose combination. The first patient responses in this set are the true $y_{ij}$ DLT responses and $n_{ij}-y_{ij}$ non‐DLT responses, in a random permutation (note that this is the same random permutation for the PIPE and BLRM). The remaining $48-n_{ij}$ responses are generated in the following way. Each patient has an individual probability of DLT, generated from $\text{Beta}(1+y_{ij},1+n_{ij}-y_{ij})$. Then a binary response is generated with this probability. Where there were no patients assigned to the dose combination in the real study, the individual probability of DLT is generated from a Beta(3,3) distribution, to indicate the dose combination is unsafe, since this is the reason the combination was not escalated to. This process uses the information from the real study, but also introduces enough variability in the subsequent responses to account for the small sample size.

Table 14 displays the results for the PIPE design (with λ=0.6) at the point of insertion, and Table 15 displays the results for the BLRM (with λ=0.8) once all 48 patients have been treated. Each entry contains the number of patients experiencing a DLT and the number of patients at each combination. The combination selected for phase II is highlighted in bold for the BLRM.

**TABLE 14:** Results for the PIPE design (with λ=0.6) applied to the case study (at the point of insertion). Each entry represents the number of patients experiencing a DLT/number of patients.

	Temsirolimus			
Neratinib	**PIPE**	25 mg	50 mg	75 mg
	120 mg	0/3	0/3	0/3
	160 mg	0/0	0/6	3/6
	200 mg	1/6	2/3	0/0

**TABLE 15:** Results for the BLRM (with λ=0.8) applied to the case study (at the end of the trial). Each entry represents the number of patients experiencing a DLT/number of patients. The combination selected for phase II is highlighted in bold.

	Temsirolimus			
Neratinib	**BLRM**	25 mg	50 mg	75 mg
	120 mg	0/3	0/3	0/3
	160 mg	0/0	**2/21**	5/9
	200 mg	1/3	5/6	0/0

Both designs begin similarly by escalating quickly through combinations consisting of the lowest dose of neratinib. Following this, the PIPE design recruits a small number of subjects to most of the remaining combinations in the grid and establishes that the (160 mg, 75 mg) and (200 mg, 50 mg) combinations are too toxic. It also estimates that combinations adjacent to these from below are less than the target toxicity. This leads to the PIPE design recommending an insertion after only 30 patients in total have been treated.

On the other hand, the BLRM ultimately leads to more patients being treated at the toxic (160 mg, 75 mg) and (200 mg, 50 mg) combinations, compared with the PIPE design. The results also demonstrate poor exploration of the grid, epitomized by the repeated testing of patients at the suspected subtherapeutic (160 mg, 50 mg) combination. Moreover, it does not explore a possible candidate for phase II further, namely the (200 mg, 25 mg) combination, despite the fact that the trial finished with one of three patients experiencing a DLT. No insertion is recommended, and the full sample size of 48 is used.

Additionally, applying the PIPE design to our case study provides a useful example of how the dosing grid changes following an insertion. Table 16 illustrates the new dosing grid directly after the insertion occurs. The underlying escalation design ensures that the combination administered directly after the insertion is comprised of at least one of the new dose levels. In Table 16, these candidates are denoted by the “+” symbols. While theoretically the number of combinations increases from nine to 25, the underlying escalation design will target future exploration around the combinations close to the target toxicity. Overdosing criteria within the underlying escalation design prohibit treating patients at new combinations that are likely overly toxic.

**TABLE 16:** Illustration of the combination space following the PIPE design recommending an insertion (with λ=0.6) applied to the case study. Each entry represents the number of patients experiencing a DLT/number of patients. Inserted dose levels are highlighted in bold. Candidates for the subsequent cohort are denoted by the “+” symbols.

	Temsirolimus					
Neratinib	**PIPE**	25 mg	**37.5 mg**	50 mg	**62.5 mg**	75 mg
	120 mg	0/3		0/3		0/3
	**140 mg**				+	+
	160 mg	0/0		0/6	+	3/6
	**180 mg**		+	+	+	
	200 mg	1/6	+	2/3		0/0

## 6 Discussion

This paper investigates potential solutions to early‐phase combination trials in oncology in which none of the initial set of combinations have a toxicity probability close to the target level. We propose a novel method to insert dose levels mid‐trial, achieved through estimation of the MTC, a concept first proposed for the PIPE design. Results from our comprehensive simulation study suggest insertions increase the probability of selecting combinations close to the target toxicity when applied to the PIPE design, without increasing the risk of making toxic selections. Applying the insertion method does not negatively impact performance in cases where an insertion is not necessarily needed.

While the paper only explores the insertion procedure applied to the PIPE design and BLRM, theoretically, the method can be coupled with any model‐based or model‐assisted design. Our work suggests the parametrization of the BLRM does not encourage effective modeling of the dose‐toxicity relationship, highlighting the importance of specifying a flexible model. Its poor underlying performance for the 3×3 dosing grid also limits the positive effects of the insertion procedure. The PIPE design is far superior in allowing wider exploration of the dosing grid, enabling more informed insertion decisions. This is reflected in the case study in Section 5.

With particular focus on the insertion procedure applied to the PIPE design, the method provides a viable alternative to the current solutions, which typically involve ad hoc decisions mid‐trial. We believe this is a useful and efficient addition to the underlying escalation design if discrete doses midway between existing doses are practically and commercially available. Possible extensions to this research include considering the implications of one treatment having a dose on the continuous scale or incorporating efficacy data into the insertion procedure.

From a practical perspective, we strongly encourage operational discussions to occur at the protocol development stage, with details of the insertion method and possible new doses to be documented in the protocol. Planning at this early stage would help mitigate the risk of delays during trial conduct, and has the advantage of eliminating the need for protocol amendments should any dose insertion occur.

We conclude our discussion with some comments from an investigator and regulatory perspective. While the process of data‐driven dose insertions requires certain adjustments to the trial processes, many of these are already in place in early‐phase dose‐escalation trials, and our proposal is to formalize these. Specifically, the majority of clinical trial design protocols for Phase I trials in which the authors of this paper are involved have the statement that “for the considered dose range the intermediate doses can be tried if prompted by the observed information.” So while no intermediate dose levels are stated in the protocol, the flexibility of testing doses in between existing dose levels is often specified, and accepted by the regulators in our experience. Since our proposal allows only for insertions within the pre‐approved minimum and maximum dose range, we know regulators are accepting of such insertions.

## Funding

Pavel Mozgunov's research is supported by the National Institute for Health and Care Research (NIHR Advanced Fellowship, Pavel Mozgunov, NIHR300576). Pavel Mozgunov received funding from UK Medical Research Council (MC UU 00040/03).

## Conflicts of Interest

The authors declare no conflicts of interest.

## Supporting information




**Data S1**: Supporting Information.

## Data Availability

The data that support the findings of this study are available from the corresponding author upon reasonable request.
