# Supplementary material for: A Novel Method for Inserting Dose Levels Mid‐Trial in Early‐Phase Oncology Combination Studies
Source: Stat Med. 2026 Feb 12;45(3-5):e70417. doi: 10.1002/sim.70417 (PMC12917875; doi:10.1002/sim.70417)
Supplement: Supplementary file 1 — Data S1: Supporting Information. [file SIM-45-0-s001.pdf]

1 | SUPPLEMENTARY MATERIALS

Following the estimation of MTC_θ, the rule in the main simulation study is to insert only if $\text{argmax}_{C_s \in \vartheta} \mathbb{P}(MTC_\theta = C_s | n_{ij}, y_{ij}) > \lambda$. However, alternative strategies determining when to insert new doses were considered.

These strategies involved estimating two other contours, which we call the underdosing and overdosing contours, in addition to estimating MTC_θ as before. These are denoted as UDC_{a_1} and ODC_{a_2} respectively for some $a_1 < \theta$ and $a_2 > \theta$. These were estimated similarly to the MTC_θ, replacing θ with a_1 and a_2 in Equation 2. Once each of the contours MTC_θ, UDC_{a_1} and ODC_{a_2} were estimated, alternative rules as described in Equations S1 and S2 were tested to see if an insertion was triggered. The rationale is that agreement in at least two of the contours strongly suggests we can partition the dose combination space into combinations with toxicity above and below θ correctly, suggesting no existing combination is close to θ.

$$\begin{aligned}
&\Big[\text{argmax}_{C_s \in \vartheta} \mathbb{P}(MTC_\theta = C_s | n_{ij}, y_{ij}) = \text{argmax}_{C_s \in \vartheta} \mathbb{P}(UDC_{a_1} = C_s | n_{ij}, y_{ij}) \\
&\quad \text{and } \mathbb{P}(MTC_\theta = C_s | n_{ij}, y_{ij}) > \lambda \text{ and } \mathbb{P}(UDC_{a_1} = C_s | n_{ij}, y_{ij}) > \lambda\Big] \\
&\qquad \text{or} \\
&\Big[\text{argmax}_{C_s \in \vartheta} \mathbb{P}(MTC_\theta = C_s | n_{ij}, y_{ij}) = \text{argmax}_{C_s \in \vartheta} \mathbb{P}(ODC_{a_2} = C_s | n_{ij}, y_{ij}) \\
&\quad \text{and } \mathbb{P}(MTC_\theta = C_s | n_{ij}, y_{ij}) > \lambda \text{ and } \mathbb{P}(ODC_{a_2} = C_s | n_{ij}, y_{ij}) > \lambda\Big] \qquad \text{(S1)}
\end{aligned}$$

$$\begin{aligned}
&\Big[\Big\{\text{argmax}_{C_s \in \vartheta} \mathbb{P}(MTC_\theta = C_s | n_{ij}, y_{ij}) = \text{argmax}_{C_s \in \vartheta} \mathbb{P}(UDC_{a_1} = C_s | n_{ij}, y_{ij})\Big\} \\
&\quad \text{and } \Big\{\mathbb{P}(MTC_\theta = C_s | n_{ij}, y_{ij}) > \lambda \text{ or } \mathbb{P}(UDC_{a_1} = C_s | n_{ij}, y_{ij}) > \lambda\Big\}\Big] \\
&\qquad \text{or} \\
&\Big[\Big\{\text{argmax}_{C_s \in \vartheta} \mathbb{P}(MTC_\theta = C_s | n_{ij}, y_{ij}) = \text{argmax}_{C_s \in \vartheta} \mathbb{P}(ODC_{a_2} = C_s | n_{ij}, y_{ij})\Big\} \\
&\quad \text{and } \Big\{\mathbb{P}(MTC_\theta = C_s | n_{ij}, y_{ij}) > \lambda \text{ or } \mathbb{P}(ODC_{a_2} = C_s | n_{ij}, y_{ij}) > \lambda\Big\}\Big] \qquad \text{(S2)}
\end{aligned}$$

Alongside the rule used in our main simulation study, we formally define four alternative insertion rules, each of which depends on either Equation S1 or S2, and a pair (a_1, a_2).

- Rule 0: Insert based on the rule used in the main simulation study.
- Rule 1a: Insert if Equation S1 is satisfied, with $(a_1, a_2) = (0.15, 0.45)$.
- Rule 1b: Insert if Equation S2 is satisfied, with $(a_1, a_2) = (0.15, 0.45)$.
- Rule 2a: Insert if Equation S1 is satisfied, with $(a_1, a_2) = (0.25, 0.35)$.
- Rule 2b: Insert if Equation S2 is satisfied, with $(a_1, a_2) = (0.25, 0.35)$.

Rule 0 is the simplest we consider, with the advantage of being easier to communicate to clinicians. Rule 1a is the strictest; since at least two of the three contours defined must be identified as the MTC with the highest probability, each of which must be greater than λ.

Tables S1 and S2 summarise the key operating characteristics for the PIPE design (using $\lambda = 0.6$) and the BLRM (using $\lambda = 0.8$) when applying different insertion rules, respectively. As with the main simulation study, we run 1000 simulations for each scenario to fully explore the behavior of our insertion procedure.

In Table S1, we observe the sensitivity of the PIPE design to changes in the insertion rule. The proportion of insertions in Rule 2a are very similar to that in Rule 0; subsequently there is minimal change to the PCS, PAS and PTS. Due to the additional strictness of Rule 1a, the proportion of insertions are reduced in all scenarios, by approximately 5% on average, resulting in lower PCS. The proportion of insertions

in Rule 2b are notably greater in all scenarios, relative to Rule 0. There is a marked increase in the PCS for the first 6 scenarios where an insertion is more desirable, and minimal change in the PCS for other scenarios. The trade-off is an increase in the PTS in some scenarios, although not considerable. Rule 1b has the greatest increase in the proportion of insertions compared to Rule 0, but this is at the cost of a spike in PTS in most scenarios, by more than 4% in six scenarios.

Similarly in Table S2, we observe the sensitivity of the BLRM to changes in the insertion rule. The proportion of insertions in Rules 1a and 2a differ by less than 1% to Rule 0 in almost all cases, leading to these rules having similar operating characteristics to Rule 0 in all scenarios. In Rule 2b we observe a notable increase in the proportion of insertions compared to Rule 0. In Scenario A1 there is a 6% increase in the PCS, although the increase in PCS across other scenarios, if any, is minimal, whilst the PTS increases slightly in scenarios which do not require an insertion. Rule 1b has a drastic increase in insertions relative to Rule 0 in most scenarios. We observe a marked increase in PCS in Scenarios A1 and B1 without a notable increase in the PTS in any scenario.

Overall, the sensitivity analysis of the rule depicts the trade-off in the operating characteristics, with insertions typically leading to higher PCS, but also higher PTS. Whilst we acknowledge the rule can have an impact, Rule 0 strikes a good balance between the operating characteristics, and is more simple to communicate than Rules 1a and 2a whose behaviours align closely.

Scenario	A1	A2	A3	B1	B2	B3	C1	C2	C3	D1	D2	D3
**Rule 0**												
Insertion (%)	48.8	36.5	39.3	38.1	31.9	25.2	31.0	25.6	32.1	24.9	22.4	25.8
PCS (%)	28.3	22.7	21.4	15.5	14.3	8.5	49.2	59.3	37.4	45.7	50.2	23.4
PAS (%)	28.3	24.6	26.9	68.0	77.5	83.3	57.5	68.7	52.2	75.2	90.6	88.8
PTS (%)	16.6	18.7	12.8	14.6	14.3	6.0	18.4	6.0	2.0	16.0	4.7	1.7
**Rule 1a**												
Insertion (%)	44.9	34.1	37.5	32.4	23.5	23.4	23.5	19.7	29.2	21.3	17.7	20.6
PCS (%)	24.8	21.6	18.7	13.6	10.2	8.4	52.7	58.0	38.5	45.1	48.3	23.1
PAS (%)	24.8	23.0	24.0	68.8	79.6	83.0	60.8	65.4	50.9	77.9	90.9	91.0
PTS (%)	16.1	18.6	13.5	13.9	13.7	5.9	12.6	6.1	1.3	14.6	4.8	1.1
**Rule 1b**												
Insertion (%)	89.0	74.6	64.4	69.5	69.7	53.8	74.3	56.9	61.1	63.1	57.6	53.1
PCS (%)	43.1	44.9	36.0	28.4	31.6	18.3	41.1	54.0	37.0	35.9	48.1	19.6
PAS (%)	43.1	48.0	43.7	67.5	74.6	80.5	63.0	76.4	65.2	67.1	85.4	85.9
PTS (%)	26.7	23.6	12.6	18.5	15.0	7.5	27.1	10.2	3.4	25.3	9.1	2.4
**Rule 2a**												
Insertion (%)	50.7	37.9	39.5	37.3	27.4	27.0	30.9	25.6	32.2	25.3	22.4	25.1
PCS (%)	27.7	25.4	19.3	13.0	13.2	10.6	49.6	56.9	36.6	47.1	48.7	20.2
PAS (%)	27.7	27.1	23.6	67.0	78.7	84.3	58.2	65.7	53.0	77.5	89.3	87.7
PTS (%)	17.1	17.3	11.9	15.9	14.1	5.9	17.0	6.9	2.6	14.7	6.8	1.2
**Rule 2b**												
Insertion (%)	76.9	49.9	43.0	61.7	42.8	30.4	49.5	31.9	35.6	43.5	35.2	29.4
PCS (%)	40.5	30.9	21.0	26.5	19.8	10.3	46.8	55.5	39.6	47.6	45.8	23.3
PAS (%)	40.5	32.9	27.1	65.7	77.5	81.4	64.7	67.3	55.9	74.7	88.5	88.5
PTS (%)	23.6	19.6	12.3	19.3	15.3	5.6	20.1	9.0	1.7	19.0	7.4	1.0

TABLE S1 Key operating characteristics for the PIPE design (with $\lambda = 0.6$) when following different insertion rules. PCS = proportion of correct selections. PAS = proportion of acceptable selections. PTS = proportion of toxic selections.

Scenario	A1	A2	A3	B1	B2	B3	C1	C2	C3	D1	D2	D3
**Rule 0**												
Insertion (%)	9.9	2.5	12.1	4.4	2.8	4.6	3.4	3.5	11.9	2.1	2.9	8.6
PCS (%)	4.8	0.9	1.7	2.0	0.4	0.5	57.7	64.1	56.4	34.7	46.5	41.2
PAS (%)	4.8	1.0	2.5	63.1	72.2	66.1	58.5	64.7	57.6	72.3	86.4	80.8
PTS (%)	25.9	38.9	41.9	9.8	21.1	22.9	8.4	10.3	15.5	4.7	8.8	11.2
**Rule 1a**												
Insertion (%)	8.7	2.3	9.3	3.4	1.9	5.1	2.6	3.5	10.4	0.5	3.0	9.0
PCS (%)	4.3	0.5	1.4	1.0	0.1	1.1	60.0	64.1	55.2	34.8	47.4	39.7
PAS (%)	4.3	0.5	1.8	63.1	74.1	66.6	60.8	64.5	56.5	69.9	84.1	78.7
PTS (%)	26.1	36.5	42.0	10.1	20.8	24.0	9.0	11.8	16.4	6.4	10.0	11.9
**Rule 1b**												
Insertion (%)	60.8	13.5	16.5	24.2	19.1	12.7	34.9	11.9	20.8	15.1	19.9	16.4
PCS (%)	35.9	3.7	1.6	11.3	3.5	2.3	57.4	62.8	56.7	33.8	47.7	38.6
PAS (%)	35.9	3.7	2.0	59.4	71.4	66.0	67.6	64.4	58.2	69.7	85.6	79.7
PTS (%)	22.0	36.6	41.7	11.4	22.3	23.5	10.1	12.3	16.9	6.7	9.9	11.9
**Rule 2a**												
Insertion (%)	9.6	3.1	9.1	3.9	2.5	4.2	3.5	4.5	11.7	2.2	3.3	8.2
PCS (%)	5.4	0.9	1.9	1.1	0.4	0.7	54.2	64.0	57.2	33.3	44.9	39.0
PAS (%)	5.4	1.0	2.1	66.1	73.6	65.5	55.6	64.7	58.4	69.7	86.0	78.9
PTS (%)	26.8	37.1	42.4	9.0	21.4	23.2	8.4	11.7	17.6	5.6	8.9	12.0
**Rule 2b**												
Insertion (%)	18.1	5.7	14.1	6.7	6.3	7.8	5.1	6.9	19.3	3.6	5.1	13.0
PCS (%)	10.7	1.6	1.7	2.4	1.1	1.7	58.5	64.6	53.3	34.9	44.2	38.5
PAS (%)	10.7	1.9	2.0	61.4	73.8	65.9	59.8	65.7	54.6	71.9	84.9	80.7
PTS (%)	23.9	36.1	43.2	9.5	18.9	24.6	11.3	10.8	17.7	5.6	9.5	11.7

TABLE S2 Key operating characteristics for the BLRM (with $\lambda = 0.8$) when following different insertion rules. PCS = proportion of correct selections. PAS = proportion of acceptable selections. PTS = proportion of toxic selections.
